# Electroretinographic recordings with skin electrodes to assess effects of vitrectomy with gas tamponade on eyes with rhegmatogenous retinal detachment

**DOI:** 10.1038/s41598-019-56307-z

**Published:** 2019-12-27

**Authors:** Masayuki Shibuya, Yuji Yoshikawa, Takeshi Katsumoto, Takuhei Shoji, Hiromi Kondo, Hitomi Miyakoshi, Kei Shinoda

**Affiliations:** Departments of Ophthalmology, Saitama Medical University, Faculty of Medicine, Saitama, Japan; 38 Moro-Hongo Moroyama-machi, Iruma-gun, Saitama 350-0495 Japan

**Keywords:** Retinal diseases, Vision disorders, Translational research

## Abstract

The purpose of this study was to evaluate the retinal function by electroretinograms (ERGs) recorded with the RETeval system using skin electrodes after pars plana vitrectomy (PPV) with gas tamponade in eyes with a rhegmatogeneous retinal detachment (RRD). Flicker ERGs were recorded from 17 eyes with an RRD before (baseline), within 2 weeks after the PPV when the size of the tamponade gas was approximately one-half of the vitreous cavity (P1), and when the gas had been completely absorbed (P2). The amplitudes of the flicker ERGs at each phase were compared to that at the baseline. The median (25th, 75th percentile) of the amplitude was 10.0 µV (5.5, 13.0 µV) at the baseline, 11.7 µV at P1 (7.8, 14.8 µV; *P* = 0.003), and 17.1 µV at P2 (11.7 23.3 μV; *P* < 0.001). The ratio of the amplitudes in the affected eye to that in the fellow eye at the baseline and at each phase was calculated, and the ratio of the amplitudes at P1 and P2 were significantly and positively correlated *(P* = 0.723, *P* = 0.001; Spearman’s rank correlation coefficient). We conclude that recordings the flicker ERGs with skin electrodes can be used to assess the physiology of eyes even with the vitreous cavity half-filled with the gas used to tamponade the retina.

## Introduction

A retinal detachment (RD) is a serious eye disorder that can lead to blindness^1,2^, and a rhegmatogenous RD (RRD) is the most common form of RD. Electroretinography (ERG) is a useful method to objectively assess the physiological status of the retina affected by vitreoretinal disorders including RRDs^3^. The ERGs can be severely decreased in eyes with an RRD, and reattachment of the retina can lead to a rapid recovery^4^.

Several authors have assessed the physiological status of the retina of eyes with an RRD before and after surgery by full-field ERGs or multifocal ERGs (mfERG)^5–12^. Many of these studies evaluated the retina after scleral buckling surgery and a fewer number of studies after pars plana vitrectomy (PPV). We investigated the effect of vitreous surgery on the ERGs of eyes with an RRD during the early postoperative period.

One difficulty in recording ERGs after PPV is the presence of intravitreal non-electrically conductive gas or silicone oil that was used to tamponade the retina. It has been reported that the intravitreal gas can alter the ERGs^5,6^, and most studies assessed the physiological condition of the retina at a later postoperative period after the gas has been absorbed. Clinicians have also been reluctant to record ERGs soon after PPV because of the fear of intraocular infections by the contact lens electrode used to record the ERGs. However, knowing the physiological status of the retina soon after the PPV would be helpful.

A new RETeval system (LKC Technologies Inc., Gaithersburg, MD; Welch Allyn, Inc., Skaneateles Falls, NY) has been developed which uses skin electrodes to pick-up the ERGs. These skin electrodes should minimize the risk of intraocular infections, and ERGs can be recorded soon after PPV to assess the physiology of the retina^13–16^. The RETeval system is a handheld, portable ERG device that uses a skin electrode to pick-up the electrical signals.

The purpose of this study was to determine whether the RETeval system can be used to record ERGs to assess the physiology of the retina soon after RRD surgery with a gas tamponade. The 30 Hz stimuli of the RETeval system elicits photopic ERGs.

## Subjects and Methods

### Subjects

All of the participants had undergone PPV with gas tamponade at the Saitama Medical University Hospital in Saitama, Japan from April 2018 to October 2018. All of the patients had signed an informed consent after the nature and possible complications of the surgery and ERG recording procedures were explained. This retrospective study was conducted in accordance with the tenets of Declaration of Helsinki, and the procedures were approved by the Ethics Committee of Saitama Medical University, Saitama, Japan (ID number: 14–122).

Twenty eyes of 20 patients with unilateral RRD were studied. The contralateral eyes were analyzed in the same way and served as normal controls. All had undergone PPV with 20% sulfur hexafluoride gas tamponade. The median follow-up period was 3 months with a range of 1 to 6 months. Patients with hereditary retinal disorders, proliferative vitreoretinopathy (PVR) grade C or higher, vascular retinal disorders, chorioretinal inflammation, media haziness, or eyes that had undergone combined encircling surgery were excluded. Three eyes were excluded because they had undergone combined encircling, which may affect the effects of vitrectomy with gas tamponade in eyes with RRD. In the end, 17 eyes were included in the statistical analyses. There were 12 men and 5 women with a median (25th, 75th percentile) age of 62 years (54, 67 years) with a range of 50 to 82 years. The duration of the symptoms, extent of the detached retina, best-corrected visual acuity (BCVA), and lens status were obtained from the patients’ medical charts.

### Surgery

All 17 patients with primary RRD underwent standard PPV. Thirteen eyes had cataracts and underwent combined cataract surgery with intraocular lens implantation, and 4 eyes were pseudophakic. After phacoemulsification and IOL implantation, 25-gauge trocars were inserted into the vitreous, and a complete posterior vitreous detachment was created by suction with a vitrectomy probe (CONSTELLATION^®^ Vision System, Alcon, Bromma, Sweden), if needed. Endoillumination was obtained from a 25-gauge standard fiberoptic system (ref. no. 8065812001) using halogen light bulbs (XENOPHOT HLX, HLX 64627 EFP 12 V 100 W, Osram, München, Germany). Triamcinolone acetonide (MaQaid®, Wakamoto, Co., Ltd, Tokyo, Japan) was used to make the vitreous gel and posterior hyaloid membrane more visible. The vitreous cutting speed was 5,000 cuts/min, and the vitreous traction was released by shaving the vitreous gel around all retinal tears and peripheral regions as close to the vitreous base as possible. Liquid perfluorocarbon was used to stabilize the detached retina if needed. Fluid-air exchange was performed and subretinal fluid was drained by internal drainage with a backflash needle (DORC). Photocoagulation was performed with an endolaser probe around breaks and around local retinal degeneration with vitreoretinal adhesion such as lattice degeneration and vitreous tag. Cryopexy was not used.

The air was exchanged for approximately 20% sulfur hexafluoride (SF_6_) to fill the vitreous cavity in all eyes. The intraocular pressure was maintained at 30 mmHg during the air and gas infusion.

The median time of surgery was 90 minutes with a range of 65 minutes to 150 minutes.

### ERG recordings

Flicker ERGs were recorded three times from both eyes with the RETeval system; the first recording was before the surgery (baseline), the second was within 2 weeks after the PPV when the size of the tamponade was approximately one-half of the vitreous cavity (Phase 1), and the third was when the tamponade gas was completely absorbed (Phase 2).

The ERG recordings were made after mydriasis under room light, and the 30 Hz flicker ERGs were picked-up by a sensor strip skin electrode affixed to the lower eyelid of both eyes. The strips included the active, reference, and ground electrodes.

A mini Ganzfeld dome was placed in front of the eye, and 3 cd·s/m^2^ flashes on a 30 cd/m^2^ background was used to elicit the flicker ERGs. The light stimulation conditions conformed to the standard recommended by the International Society for Clinical Electrophysiology of Vision (ISCEV)^17^. The patients were instructed to look at a fixation point within the dome, and the patient’s fixation was monitored by an infrared camera. The implicit times and amplitudes of the flicker ERGs were automatically analyzed by the software integrated in the RETeval system. The ratio of the amplitudes and implicit times of the affected eye to that of the healthy fellow eye at the baseline was calculated.

### Statistical analyses

The ratios, the affected eye/normal fellow eye, of the amplitudes and implicit times at each phase were compared to that at baseline using the Wilcoxon signed rank test. The decimal BCVAs were converted to logarithm of minimum angle of resolution (logMAR), and the significance of the differences between the before and after surgery was determined with the Wilcoxon signed rank test. The BCVAs of ‘counting fingers’, ‘hand movements’, ‘light perception’ were assigned values of 2.0, 2.4, and 2.7 logMAR units, respectively (Schulze-Bonsel *et al*.)^18^. All data are expressed as the medians and the 25^th^ and 75^th^ percentile. A *P*-value of <0.05 was considered statistically significant. All statistical analyses were performed using JMP version 10.1 software (SAS Institute Inc., Cary, NC, USA).

### Patient consent

Written consent to publish this case has not been obtained. This report does not contain any personal identifying information.

## Results

The demographics and clinical data of the patients are summarized in Table 1. Fourteen eyes were phakic and 3 were pseudophakic at the time of the PPV. An RRD involving macula (macular off) was found in 10 eyes and RRD not involving the macula (macular on) was found in 7 eyes. None of the eyes had a macular hole and one eye had epiretinal membrane which was peeled off during the surgery. A posterior vitreous detachment did not exist in 2 of 17 eyes, and a posterior vitreous detachment was created in these two eyes during surgery. All retinas of the affected eyes were successfully reattached, and the retinas remained anatomical attached during the entire follow-up period. The median (25th, 75th percentile) duration of the symptoms was 3.0 days (3.0, 6.0) with a range of 1 to 18 days. No significant postoperative complications were seen.

**Table 1:** Demographics of the patients and eyes with RRD.

Number of eyes	
Age (year)*	62 [54, 67] (50 to 82)
Sex (male/female)	12/5
**Extent of detached area (eyes)**	
1 or 2 qds.	13
3 or 4 qds.	4
**Dominance of the detached area (eyes)**	
superior	8
inferior	5
temporal or nasal	4
Number of tears*	2 [1,3] (1 to 5)
Duration of symptoms before surgery (days)*	3.0 [3.0, 6.0] (1 to 18)
**Visual acuity (logMAR)#**	
before surgery*	0.40 [0.20, 1.85] (−0.08 to 2.0)
after surgery*	0.00 [−0.08, 0.05] (−0.08 to 0.40)

*Data are expressed as median [25th, 75th percentile] (range). RRD: rhegmatogenous retinal detachment qds: quadrants log MAR:logarythm of minimal angular resolution^#^.counting finger was assigned as 2.0 of logMAR.

The median time when the ERG recordings were made was 7 days (5, 10 days) with a range of 4 to 12 days, and the median time when the ERGs were recorded postoperatively was 27 days (19, 34 days) with a range of 13 to 53 days. No significant correlation was found between ‘the ERG changes’ and ‘the interval between the time of the surgery and the ERG recordings’ (*P* = 0.66 for ‘the ERG change from baseline to P1’ vs ‘the interval from surgery to P1’, *P* = 0.76 for ‘the ERG change from baseline to P2’ vs ‘the interval from surgery to P2’).

The median BCVA before surgery was 0.40 logMAR units (0.20, 1.85 logMAR units) with a range of −0.08 to 2.0 logMAR units. The BCVA improved significantly to 0.00 logMAR units (−0.08, 0.05 logMAR units) with a range of −0.08 to 0.40 logMAR units at the final follow-up examination (*P < *0.001).

The median amplitude of the affected eye was 10.00 µV (5.5, 13.0 µV) with a range of 1.6 to 21.2 µV at the baseline, 11.7 µV (7.8, 14.8 µV) with a range of 0.0 to 28.4 µV (*P* = 0.003) at Phase 1, and 17.1 µV (11.7, 23.3 µV) with a range of 3.1 to 32.5 μV (*P < *0.001) Phase 2 **(**Table 2**)**. The median implicit time was 25.8 ms (24.7, 26. ms) with a range of 24.2 to 30.5 ms at the baseline, 27.2 ms (26.2, 28.5 ms) with a range of 24.2 to 31.0 ms at Phase 1, and 27.2 ms (27.0, 29.0 ms) with a range of 26.2 to 30.7 ms at Phase 2 **(**Table 2**)**.

**Table 2:** The amplitude and the implicit time of flicker electroretinogram in the affected and the healthy fellow eye.

	affected eye			fellow eye at baseline as control	a/c ratio		
	Baseline	Phase 1	Phase 2		Baseline	Phase 1	Phase 2
Amplitude (μV)	10.0 (5.5, 13.0) (range; 1.6 to 21.2)	11.7 (7.8, 14.8) (range; 0.0 to 28.4)	17.1 (11.7, 23.3) (range; 3.1 to 32.5)	26.5 (17.4, 30.0) (range; 9.9 to 33.5)	0.37 (0.30, 0.50) (range; 0.00 to 0.74)	0.51 (0.38, 0.65) (range; 0.00 to 0.99)	0.75 (0.55, 1.04) (range; 0.10 to 1.62)
Implicit time (msec)^#^	25.8 (24.7, 26.4) (range; 24.2 to 30.5)	27.2 (26.2, 28.5) (range; 24.2 to 31.0)	27.2 (27.0, 29.0) (range; 26.2 to 30.7)	25.5 (25.1, 26.1) (range; 24.8 to 26.9)	1.00 (0.97, 1.04) (range;0.95 to 1.16)	1.07 (1.02, 1.10) (range; 0.92 to 1.18)	1.07 (1.05, 1.09) (range; 0.99 to 1.17)

*Data are expressed as median (25th, 75th percentile) (range).

^#^The eye showing noise level response was excluded from the calculation since the implicit time was not measurable.

**Figure 1:**
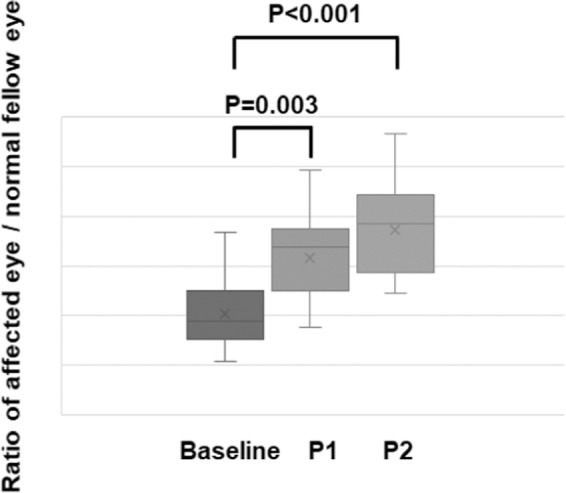
Ratio of the amplitude of an eye with a rhegmatogenous retinal detachment to that of the normal fellow eye at the baseline, at Phase 1, and Phase 2. The ratio of the amplitudes is significantly larger at Phase 1 and Phase 2 compared to that at the baseline. The error bars represent the standard error of the means (SEMs) and the boxes represent the median (quantiles). “X” represents the mean value. Statistical analyses were done by Wilcoxon signed rank tests; the ratio was calculated as the amplitude of the affected eye to that of the control eye. Each value is shown in Table 2. RRD, rhegmatogenous retinal detachment; P1, Phase 1; P2, Phase 2.

The ratio of the median amplitudes of the flicker ERGs in the affected eyes to that of the fellow eyes was 0.37 (0.30, 0.50) with a range of 0.00 to 0.74 at the baseline, 0.51 (0.38, 0.65) with a range of 0.00 to 0.99 (*P* = 0.003) at Phase 1, and 0.75 (0.55, 1.04) with a range of 0.10 to 1.62; *P* < 0.001) at Phase 2 **(**Fig. 1**)**. The increase of the ratio indicated that the amplitudes increased significantly after the surgery.

The ratio of the median implicit times of the affected eye to that in the fellow eye was 1.00 (0.97, 1.04) with a range of 0.95 to 1.16 at the baseline, 1.07 (1.02, 1.10) with a range of 0.92 to 1.18 at Phase 1, and 1.07 (1.05, 1.09) with a range of 0.99 to 1.17 at Phase 2. In cases where the amplitudes of the ERGs were at noise level, the amplitudes were set to 0 µV and the implicit time was unmeasurable. Thus, the implicit times were not suitable for statistical analyses, and statistical analyses was only performed on the amplitudes.

**Figure 2:**
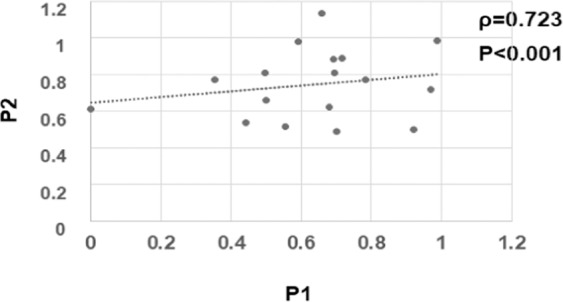
Distributions of the ratios (affected eye/normal fellow eye) of the amplitudes of the flicker electroretinograms at Phase 1 and Phase 2. The ratio of the amplitude was calculated as the ratio of the amplitude of the affected eye to that of the normal fellow eye. The distribution of the ratios of the amplitudes in eyes with rhegmatogenous retinal detachment at Phase 2 against that at Phase 1 is plotted. The ratio at Phase 1 was significantly and positively correlated to the ratio at Phase 2 (*rho* = 0.723, *P* = 0.001, Spearman’s correlation coefficient).

There was a significant positive correlation between the ratios at Phase 1 and Phase 2 (Fig. 2, *ρ* = 0.723, *P* = 0.001; Spearman’s rank correlation coefficient).

**Figure 3:**
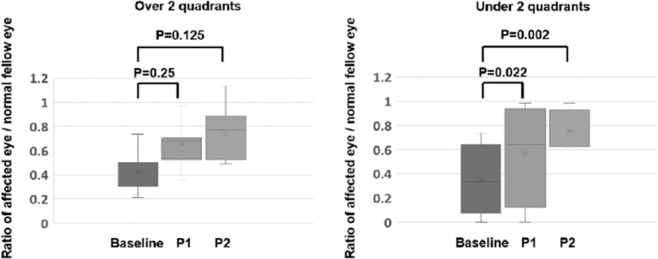
Ratios (affected eye/normal fellow eye) of the amplitudes of the flicker electroretinograms in eyes with RRD area greater than (n = 4) or less than (n = 13) 2 quadrants are plotted. The ratios of the amplitudes of the 2 groups that were classified according to the size of the rhegmatogenous retinal detachment area as either >2 quadrants (n = 4) or ≤2 quadrants (n = 13) were compared. The ratios of the amplitudes in eyes with rhegmatogenous retinal detachment (RRD) area greater than (left) and less than 2 quadrants (right) at the baseline, Phase 1, and Phase 2 is shown. The increase in P1 was significant in eyes with an RRD area ≤2 quadrants (left) but not in eyes with RRD area >2 quadrants (right). Error bar represents the SEMs and boxes represent median (quantiles). X represents the mean value. Statistical analyses were done by Wilcoxon signed rank tests.

The ratios of the amplitudes of eyes in which the RRD extended for ≤2 quadrants (n = 13) were compared to the eyes in which the RRD extended >2 quadrants (n = 4, Fig. 3). At P1, the ratio of the amplitudes was significantly larger than that at the baseline in eyes with RRD area was ≤2 quadrants (*P* = 0.022), but not in eyes with RRD area >2 quadrants (*P* = 0.25). The significant increase in the ratio at P2 compared to baseline was observed in eyes with RRD area <2 quadrants (*P* = 0.002) and not eyes with RRD area >2 quadrants (*P* = 0.13).

**Figure 4:**
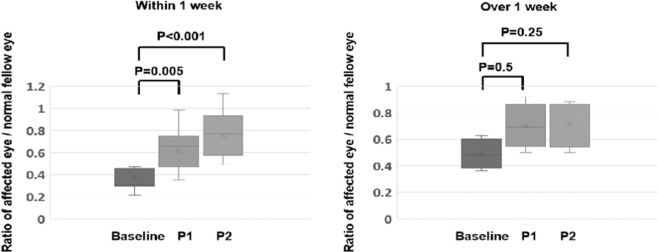
Ratios of the amplitudes of the flicker electroretinograms in eyes with rhegmatogenous retinal detachment duration less than (n = 13) or more than (n = 4) one week. The ratios of the amplitudes of the 2 groups that were classified according to the duration of the rhegmatogenous retinal detachment (RRD) either ≤1 week (n = 13) or >1 week (n = 4) are compared. The ratio in the amplitude in eyes with rhegmatogenous retinal detachment (RRD) duration less than (left) or more than one week (right) at the baseline, Phase 1, and Phase 2. The increase in the ratio of at P1 and P2 was significant in eyes with RRD duration less than one week (left), but not in eyes with RRD duration more than one week (right). Error bars are the SEMs and boxes represent the median (quantiles). “X” represents the mean value. Statistical analysis was done by Wilcoxon signed rank test.

The ratio of the amplitudes in eyes with an RRD duration of ≤1 week (n = 13) were compared to those with a duration >1 week **(**n = 4, Fig. 4**)**. The ratio of the amplitudes significantly increased at P1 and P2 compared to the baseline in eyes with RRD of ≤1 week *(P* < 0.001 for both), but not in eyes with RRD duration >1 week *(P* = 0.5 for P1 *and P* = 0.25 for P2).

## Discussion

Many authors have used ERGs to assess the outcome of reattachment surgery in eyes with a RRD, but most of these studies were on eyes that had undergone scleral buckling surgery^5,6,8–12^. Our patients had undergone PPV, and we recorded the ERGs of eyes that were one-half filled with 20% SF as well as from eyes after the gas had been completely absorbed. The SF is non-conductive and can alter the ERGs recorded^19,20^. The findings confirm the report by Miyake and Horiguchi that ERG recordings can be made after vitreous surgery with and without the presence of the gas used to tamponade the retina^21^. Our results showed that we were able to record ERGs from eyes in which the gas tamponade half-filled the vitreous cavity.

Our results showed that the amplitudes of the ERGs recorded from the one-half gas-filled eyes were significantly and positively correlated with the amplitudes after the gas had been completely absorbed. This indicates that the amplitudes of the flicker ERGs when the eye is one-half filled can be used to predict the amplitudes of the flicker ERGs when the gas has been completely absorbed. This is clinically important because these ERGs were recorded during the very early postoperative periods, and they can provide useful information about the physiological status of the retina even when ophthalmoscopy and ultrasonography cannot be used to assess the retina. For example, early postoperative vitreous hemorrhage or relatively severe inflammation with fibrin in the anterior chamber hampers fundoscopic examination, and B-mode echo evaluation is not reliable due to tamponade gas. But the ERGs can provide information on the status of the retinal function even during the early phase of recovery. In addition, when the surgeon discusses the time and prognosis of a second surgery, this information will be valuable.

Because ERG recordings takes only a few minutes, we believe that it is practical for the clinical use to obtain clinically relevant information. Frumar *et al*.^5,6^ recorded mixed rod and cone ERGs before and 3 days and 1, 3, and 6 months after vitrectomy with intraocular gas (mixture of 20% SF_6_ and 80% air) from 9 eyes with an RRD and one eye with a macular hole. Although statistical analysis was not performed, they reported that the mean amplitude of the a-wave before surgery was 51 µV which was reduced to 14 µV at 3 days but then increased to 72 µV at one month after the surgery. For the b-wave, the amplitude was 107 µV before the surgery, 48 µV at 3 days, and 137 µV at one month after the surgery. Frumar *et al*. reported that it was possible to record large ERGs even in the presence of a large body of non-conductive material in the vitreous cavity, and they suggested that there was a thin but significant conductive path between the retinal surface and the cornea which allowed the recording of the ERGs. Our results agree with their findings that sizable ERGs can be recorded from eyes half-filled with gas.

Another of our significant findings was that the amplitudes of the flicker ERGs at Phase 1 were positively and significantly correlated with the amplitudes at Phase 2. The ERGs from the gas-filled eyes in our patients were smaller than that before surgery of Frumar *et al*. studies^5,6^, and significant improvement was observed even from the gas-filled eyes. This discrepancy may be because 60 to 70% of the vitreous cavity was filled with gas in their study, but in our study about 50% was occupied by gas. Our first postoperative recordings were performed approximately 7 days after the surgery when a functional recovery of the reattached retina might be larger than at 3 days after the surgery.

The recovery of the ERGs at Phases 1 was significant in eyes with 2 or less quadrants of detachment and not in eyes with over 3 quadrants of detachment. This suggests that the functional recovery of the reattached retina may be attained within 2 weeks after surgery, but not in eyes with more extensive detachments. The ERG recovery at Phase 1 was significant in eyes with RRD duration within 7 days but not over 8 days suggesting that retinal reattachment for as long as 7 days can lead to rapid functional improvements with a reattachment of the retina.

This study has several limitations. First, only flicker ERGs were recorded because it required only a few minutes to perform. This minimized the discomfort and inconvenience for the patient. Previous studies recorded the recovery of the rods and cones^8,10–12^ and that of the outer and inner retinal layer^5,6,12^. This was not possible by our instrument. However, our results suggest that flicker ERGs can provide informative and practical information. Another limitation is that the sample size was small. Although we were able to obtain statistically significant observations, a future study with a larger sample size on eyes that had undergone the same surgical procedures should provide more appropriate information. Third, full-field ERGs do not reflect the macular function, and multifocal ERG or focal macular ERG recorded with skin electrodes would allow investigations of the relationship between macular function and the recovery of the macular are. Fourth, the Phase 2 ERG recordings were done at about 30 days after the vitreoretinal surgery. The short follow-up period may limit the conclusions. Because the minimum duration for photoreceptor recovery after retinal reattachment was reported to be four weeks^22,23^, it might be that the photoreceptors had not completely recovered. Because several ERG studies have reported that severe reduction of ERGs caused by the RRD recovered shortly after reattachment surgery with maximum recovery at 3 months^5,6,12^, another ERG recording at 3 months would be interesting. In the current study, we focused on the early postoperative periods, but we believe that another long-time follow up study will be meaningful. Fifth, the effect of 1/2 filled SF_6_ on ERG data cannot be denied^5,6^ and needs to be determined. Although not practical, the ERG analysis in eyes with intact retina and where the precise amount (%) of intravitreal SF_6_ gas is known, would answer this question.

In conclusion, our results indicate that skin electrodes with the RETeval system can be used to assess the physiological status eyes half-filled with SF_6_ gas. The findings can provide information on the degree of recovery of the retina a short time after the PPV. This is significant because the amplitudes of the ERGs at Phase 1 were significantly correlated with the ERGs at Phase 2.

## Data Availability

The datasets used and/or analyzed during the current study are available from the corresponding author on reasonable request.
