# Author Correction: Electroretinographic recordings with skin electrodes to assess effects of vitrectomy with gas tamponade on eyes with rhegmatogenous retinal detachment

**DOI:** 10.1038/s41598-020-61545-7

**Published:** 2020-03-06

**Authors:** Masayuki Shibuya, Yuji Yoshikawa, Takeshi Katsumoto, Takuhei Shoji, Hiromi Kondo, Hitomi Miyakoshi, Kei Shinoda

**Affiliations:** Departments of Ophthalmology, Saitama Medical University, Faculty of Medicine, Saitama, Japan, 38 Moro-Hongo Moroyama-machi, Iruma-gun, Saitama 350-0495 Japan

Correction to: *Scientific Reports* 10.1038/s41598-019-56307-z, published online 27 December 2019

This Article contains errors in the Subjects and Methods section under subheading ‘Subjects’.

“All of the participants had undergone PPV with gas tamponade at the Saitama Medical University Hospital in Saitama, Japan from April 2018 to October 2018. All of the patients had signed an informed consent after the nature and possible complications of the surgery and ERG recording procedures were explained. This retrospective study was conducted in accordance with the tenets of Declaration of Helsinki, and the procedures were approved by the Ethics Committee of Saitama Medical University, Saitama, Japan (ID number: 14–122).”

should read:

“All of the participants had undergone PPV with gas tamponade at the Saitama Medical University Hospital in Saitama, Japan from May 2018 to October 2018. All of the patients had signed an informed consent after the nature and possible complications of the surgery and ERG recording procedures were explained. This retrospective study was conducted in accordance with the tenets of Declaration of Helsinki, and the procedures were approved by the Institutional Review Board of Saitama Medical University, Saitama, Japan (ID number: 18133.01).”

